# The Collegiate Year in Medicine

**Published:** 1913-09

**Authors:** 


### The Collegiate Year in Medicine

Referring to our notice in the July issue that the University of Buffalo would make this requirement beginning with the session of 1914-15 and would establish its own preparatory course, the report of the joint committee of the Association of American Medical Colleges and of the A. M. A., (*Journal of the A. M. A.,* June 21, 1913), deserves careful attention. Of the 109 medical schools giving degrees, 32 already require two or more years of college study and several the entire college course. Six more will require two years college study by 1915. Twenty-four others require one year of college study and seventeen will have this requirement in 1914. Twelve states will have required at least one year of college study of medical matriculants of the class of 1918 (entering the medical school in the fall of 1914 and five already require two years of college study of this or even earlier classes.

As we have received some criticisms of the unreasonableness of requiring more than the high school course, these statistics are of value in showing that it is "a condition and not a theory that confronts us." It should be clearly understood that the criterions of the past do not apply to the present. It is no reflection on those of us who have gotten our education in the little red school house or who are self-educated, that modern requirements impose standards in accordance with facilities freely furnished at present and available only to a small minority of the youth of a generation or two ago.

It is held by some that higher requirements militate against the poor boy whose ambition is to enter a profession. This is undoubtedly true, but, so far as our own profession is concerned, the opinion is nearly unanimous that, unless standards of license are raised, the poor boy will become still poorer as a physician and that only the rich can afford to enter upon a medical career. Solely as an economic necessity to regulate supply in accordance with demand, some process of selection must be applied to those who desire to enter upon medical practice. If anyone can suggest a fairer or more appropriate, or more practical method than by raising the educational standards, we shall be glad to hear it.

Personally, with growing experience, we appreciate more and more the seriousness back of George Ade's comic story of the high brow who, in course of time, mislaid his college diploma. Sometimes we become disgusted with formal systems of education and still more so with their products, and regret the passing of the little red school house and the self-educated man. But, as a nation, we have committed ourselves definitely to the policy of free education, of increasing degrees. There are always individuals to whom existing standards do not apply and, with the increasing tendency toward formal requirements and system, such individuals must suffer. But, this fact must be remembered, that, speaking broadly, the type of man who, in the past, educated himself by the light of a pine knot, who held a book in one hand and drove a team or worked a bellows with the other, is, except by slight modification to conform to modern developments, the same that now struggles, somehow—and on the whole with less effort and greater effectiveness—to obtain the institutional training required by law, or by rules of employment.

